# Supplementary material for: A Scoping Literature Review of the Relation between Nutrition and ASD Symptoms in Children
Source: Nutrients. 2022 Mar 26;14(7):1389. doi: 10.3390/nu14071389 (PMC9003544; doi:10.3390/nu14071389)
Supplement: Supplementary file 1 [file nutrients-14-01389-s001.zip › File S1_search_strategy.pdf]

Supplementary File S1: Search strategy.

OVID Embase

#1
('dietary pattern*' OR 'dietary intake*' OR 'food intake*' OR 'food pattern*' OR 'low energy diet*' OR 'high energy diet*' OR 'healthy diet*' OR 'prudent diet*' OR 'mediterranean diet*' OR 'western diet*' OR 'traditional diet*' OR 'fast food*' OR 'junk food*' OR snack OR snacks OR nut OR nuts OR fruit OR fruits OR vegetable OR vegetables OR legume OR legumes OR 'vegetarian diet*' OR 'vegan diet*' OR 'diet quality' OR 'dietary quality' OR glycaemic index* OR 'mind diet*' OR 'nordic diet*' OR 'calorie restriction*' OR 'caloric restriction*' OR 'high caloric' OR 'high calorie*' OR nutrition OR vitamin OR zinc OR iron OR selenium OR copper OR gluten OR folate OR folates OR 'folic acid*' OR thiamine OR thiamines OR flavonoid OR flavonoids OR carotenoid OR carotenoids OR 'fatty acid' OR 'fatty acids' OR 'omega-3' OR 'omega-6' OR pufa OR lcpufa OR mufa OR 'fish oil*' OR magnesium OR iodine OR 'nutrient deficienc*' OR 'nutritional supplement*' OR 'dietary supplement*').ab,ti.

#2
exp food/ or exp diet/

#3
(child or children or childhood or kid or kids or girl* or boy* or teenage* or youth* or youngster* or preschool* or kindergarten* or 'elementary school' or juvenile* or minors or minor or childhood or pediatric* or paediatric* or schoolchild* or infant or infants or infancy or toddler* or teen or teens).ab,ti.
#4
exp child/

#5
exp anxiety disorder/ or exp autism/ or exp behavior disorder/ or exp learning disorder/ or exp mood disorder

#6
('mental disorder*' or 'mental health disorder*' or 'mental illness*' or 'psychiatric disorder*' or 'affective disorder*' or psychopathology or 'psychiatric diagnoses' or 'psychiatric diagnosis' or 'psychiatric illness*' or 'child developmental disorder*' or 'behavior disorder' or behaviour disorder* or 'externalizing disorder*' or 'internalizing disorder*').ab,ti.

#7
(depression or depressions or depressive or 'bipolar disorder*' or 'unipolar disorder* or melancholia or melancholias or melancholic or 'mood disorder*' or 'affective disorder*' or 'manic depressive disorder*' or dysthymia or dysthymic or cyclothymia or cyclothymic or 'mood disorder').ab,ti.

#8
(anxiety or anxieties or phobia or phobias or phobic or 'panic disorder*' or 'obsessive compulsive disorder*' or 'posttraumatic stress disorder' or PTSD).ab,ti.

#9

(dyslexi* OR dyscalcul* OR dysgraphia* OR agraphia* OR 'academic skill disorder*' OR 'academic skills disorder*' OR 'scholastic skill disorder*' OR 'learning difficult*' OR 'learning disorder*' OR 'learning disabilit*' OR 'learning deficien*' OR 'learning problem*' OR 'reading abilit*' OR 'reading

accuracy' OR 'reading comprehension' OR 'reading disorder*' OR 'reading disabilit*' OR 'reading difficult*' OR 'reading deficien*' OR 'reading fluency' OR 'reading problem*' OR 'reading speed*' OR OR 'math abilit*' OR 'mathematical abilit*' OR 'math accurac*' OR 'mathematical accurac*' OR 'math disorder*' OR 'mathematical disorder' OR 'math disabilit*' OR 'mathematical disabilit*' OR 'math difficult*' OR 'mathematical difficult*' OR 'math problem*' OR 'mathematical problem*' OR 'math fluency' OR 'spelling abilit*' OR 'spelling accurac*' OR 'spelling comprehension' OR 'spelling disorder*' OR 'spelling disabilit*' OR 'spelling difficult*' OR 'spelling deficien*' OR 'spelling fluenc*' OR 'spelling problem*' OR 'spelling speed' OR 'writing abilit*' OR 'writing accurac*' OR 'writing comprehension' OR 'writing disorder*' OR 'writing disabilit*' OR 'writing difficult*' OR 'writing deficiency' OR 'writing fluency' OR 'writing problem*' OR 'writing speed*').ab,ti.

#10
('attention deficit disorder*' or 'attention deficit hyperactivity disorder*' or ADHD or 'hyperkinetic syndrome' or 'hyperactiv*' or hyperkinesis or 'Oppositional Defiant Disorder').ab,ti.

#11
('pervasive development disorder*' or 'pervasive developmental disorder*' or PDD or PDDs or ASD or ASDs or autis* or Asperger* or 'autism spectrum disorder*' or kanner* or rett*).ab,ti.

#12
1 or 2

#13
3 or 4

#14
5 or 6 or 7 or 8 or 9 or 10 or 11

#15
12 and 13 and 14

#16
limit 15 to (conference abstract or 'conference review')

#17
15 not 16

Pubmed

#1
((diet[MeSH Terms]) OR food[MeSH Terms]) OR dietary pattern*[Title/Abstract]) OR dietary intake*[Title/Abstract]) OR food intake*[Title/Abstract]) OR food pattern*[Title/Abstract]) OR low energy diet*[Title/Abstract]) OR high energy diet*[Title/Abstract]) OR healthy diet*[Title/Abstract]) OR prudent diet*[Title/Abstract]) OR mediterranean diet*[Title/Abstract]) OR western diet*[Title/Abstract]) OR traditional diet*[Title/Abstract]) OR fast food*[Title/Abstract]) OR junk food*[Title/Abstract]) OR snack[Title/Abstract]) OR snacks[Title/Abstract]) OR nut[Title/Abstract]) OR nuts[Title/Abstract]) OR fruit[Title/Abstract]) OR fruits[Title/Abstract]) OR vegetable[Title/Abstract]) OR vegetables[Title/Abstract]) OR legume[Title/Abstract]) OR legumes[Title/Abstract]) OR vegetarian diet*[Title/Abstract]) OR vegan diet*[Title/Abstract]) OR "diet quality"[Title/Abstract]) OR "dietary quality"[Title/Abstract]) OR glycaemic index*[Title/Abstract]) OR mind diet*[Title/Abstract]) OR

nordic diet*[Title/Abstract]) OR calorie restriction*[Title/Abstract]) OR caloric restriction*[Title/Abstract]) OR "high caloric"[Title/Abstract]) OR high calorie*[Title/Abstract]) OR nutrition[Title/Abstract]))

OR

((((((((((((((((((((((((((((((vitamin[Title/Abstract]) OR zinc[Title/Abstract]) OR iron[Title/Abstract]) OR selenium[Title/Abstract]) OR copper[Title/Abstract]) OR gluten[Title/Abstract]) OR folate[Title/Abstract]) OR folates[Title/Abstract]) OR folic acid*[Title/Abstract]) OR thiamine[Title/Abstract]) OR thiamines[Title/Abstract]) OR flavonoid[Title/Abstract]) OR flavonoids[Title/Abstract]) OR carotenoid[Title/Abstract]) OR carotenoids[Title/Abstract]) OR "fatty acid"[Title/Abstract]) OR "fatty acids"[Title/Abstract]) OR "omega-3"[Title/Abstract]) OR "omega-6"[Title/Abstract]) OR "n-3"[Title/Abstract]) OR "n-6"[Title/Abstract]) OR pufa[Title/Abstract]) OR lcpufa[Title/Abstract]) OR mufa[Title/Abstract]) OR fish oil*[Title/Abstract]) OR magnesium[Title/Abstract]) OR iodine[Title/Abstract]) OR nutrient deficienc*[Title/Abstract]) OR nutritional supplement*[Title/Abstract]) OR dietary supplement*[Title/Abstract]))

#2
((((((((mental disorder*[Title/Abstract] OR mental health disorder*[Title/Abstract] OR mental illness*[Title/Abstract] OR psychiatric disorder*[Title/Abstract] OR affective disorder*[Title/Abstract] OR psychopathology[Title/Abstract] OR psychiatric diagnoses[Title/Abstract] OR psychiatric diagnosis[Title/Abstract] OR psychiatric illness*[Title/Abstract] OR child development disorder* [Title/Abstract] OR behavior disorder* [Title/Abstract] OR behaviour disorder* [Title/Abstract] OR externalizing disorder* [Title/Abstract] OR internalizing disorder* [Title/Abstract] OR neurodevelopmental disorders [MeSH terms])))

OR

(Depression[MeSH Terms] OR Depression[Title/Abstract] OR Depressions[Title/Abstract] OR depressive[Title/Abstract] OR bipolar disorder*[Title/Abstract] OR unipolar disorder*[Title/Abstract] OR melancholia[Title/Abstract] OR melancholias[Title/Abstract] OR melancholic[Title/Abstract] OR manic-depressive disorder*[Title/Abstract] OR dysthymia[Title/Abstract] OR dysthymic[Title/Abstract] OR cyclothymia[Title/Abstract] OR "cyclothymic"[Title/Abstract] OR Mood Disorders[MeSH Terms] OR mood disorder*[Title/Abstract]))

OR

(Anxiety Disorders[MeSH Terms] OR Anxiety[Title/Abstract] OR Anxieties[Title/Abstract] OR phobia[Title/Abstract] OR phobias[Title/Abstract] OR phobic[Title/Abstract] OR panic disorder*[Title/Abstract] OR obsessive compulsive disorder*[Title/Abstract] OR posttraumatic stress disorder*[Title/Abstract] OR "PTSD"[Title/Abstract] OR post-traumatic stress disorder*[Title/Abstract] OR stress disorders, posttraumatic[MeSH Terms]))

OR

(dyslexi* [Title/Abstract] OR dyscalcul* [Title/Abstract] OR dysgraphia* [Title/Abstract] OR agraphia* [Title/Abstract] OR academic skill disorder* [Title/Abstract] OR academic skills disorder* [Title/Abstract] OR scholastic skill disorder* [Title/Abstract] OR learning difficult* [Title/Abstract] OR learning disorder* [Title/Abstract] OR learning disabilit* [Title/Abstract] OR learning deficien* [Title/Abstract] OR learning problem*[Title/Abstract] OR reading abilit* [Title/Abstract] OR reading accuracy [Title/Abstract] OR "reading comprehension" [Title/Abstract] OR reading disorder* [Title/Abstract] OR reading disabilit* [Title/Abstract] OR reading difficult* [Title/Abstract] OR reading deficien* [Title/Abstract] OR "reading fluency" [Title/Abstract] OR reading problem* [Title/Abstract] OR reading speed* [Title/Abstract] OR math difficult* [Title/Abstract] OR mathematical difficult* [Title/Abstract] OR math abilit* [Title/Abstract] OR mathematical abilit*

[Title/Abstract] OR math accurac* [Title/Abstract] OR mathematical accurac* [Title/Abstract] OR math disorder* [Title/Abstract] OR mathematical disorder* [Title/Abstract] OR math disabilit* [Title/Abstract] OR mathematical disabilit* [Title/Abstract] OR math problem* [Title/Abstract] OR mathematical problem* [Title/Abstract] OR "math fluency" [Title/Abstract] OR spelling abilit* [Title/Abstract] OR spelling accurac* [Title/Abstract] OR "spelling comprehension" [Title/Abstract] OR spelling disorder* [Title/Abstract] OR spelling disabilit* [Title/Abstract] OR spelling difficult* [Title/Abstract] OR spelling deficien* [Title/Abstract] OR spelling fluenc* [Title/Abstract] OR spelling problem* [Title/Abstract] OR "spelling speed" [Title/Abstract] OR writing abilit* [Title/Abstract] OR writing accurac* [Title/Abstract] OR "writing comprehension" [Title/Abstract] OR writing disorder* [Title/Abstract] OR writing disabilit* [Title/Abstract] OR writing difficult* [Title/Abstract] OR "writing deficiency" [Title/Abstract] OR "writing fluency" [Title/Abstract] OR writing problem* [Title/Abstract] OR writing speed* [Title/Abstract] OR Agraphia [MeSH terms]))

OR

(attention deficit disorder* [Title/Abstract] OR attention deficit hyperactivity disorder* [Title/Abstract] OR ADHD [Title/Abstract] OR "hyperkinetic syndrome" [Title/Abstract] OR hyperactiv* [Title/Abstract] OR hyperkinesis [Title/Abstract] OR "Oppositional Defiant Disorder*" [Title/Abstract]))

OR

(pervasive development disorder* [Title/Abstract] OR pervasive developmental disorder* [Title/Abstract] PDD [Title/Abstract] OR PDDs [Title/Abstract] OR ASD [Title/Abstract] OR ASDs [Title/Abstract] OR autis* [Title/Abstract] OR Asperger*[Title/Abstract] OR autism spectrum disorder* [Title/Abstract] OR Kanner* [Title/Abstract] OR Rett* [Title/Abstract]))

#3
(child [Title/Abstract] OR children [Title/Abstract] OR childhood [Title/Abstract] OR kid [Title/Abstract] or kids [Title/Abstract] OR girl* [Title/Abstract] OR boy* [Title/Abstract] OR teenage* [Title/Abstract] OR youth* [Title/Abstract] OR youngster* [Title/Abstract] OR preschool* [Title/Abstract] OR pre-school* [Title/Abstract] OR kindergarten* [Title/Abstract] OR "elementary school" [Title/Abstract] OR elementary-school [Title/Abstract] OR juvenile* [Title/Abstract] OR minors [Title/Abstract] OR minor [Title/Abstract] OR pediatric* [Title/Abstract] OR paediatric* [Title/Abstract] OR schoolchild*[Title/Abstract] OR infant [Title/Abstract] or infants [Title/Abstract] or infancy [Title/Abstract] OR toddler*[Title/Abstract] OR teen [Title/Abstract] OR teens [Title/Abstract] OR child, preschool [MeSH terms] OR child [MeSH terms] OR infant [MeSH terms] OR infant, newborn [MeSH terms])

#1 AND #2 AND #3

Web of science

#1
(TS = ("dietary pattern*" OR "dietary intake*" OR "food intake*" OR "food pattern*" OR "low energy diet*" OR "high energy diet*" OR "healthy diet*" OR "prudent diet*" OR "mediterranean diet*" OR "western diet*" OR "traditional diet*" OR "fast food*" OR "junk food*" OR snack OR snacks OR nut OR nuts OR fruit OR fruits OR vegetable OR vegetables OR legume OR legumes OR "vegetarian diet*" OR "vegan diet*" OR "diet quality" OR "dietary quality" OR "glycaemic index*" OR "mind diet*" OR "nordic diet*" OR "calorie restriction*" OR "caloric restriction*" OR "high caloric" OR "high calorie*" OR nutrition OR vitamin OR zinc OR iron OR selenium OR copper OR gluten OR folate OR folates OR "folic acid*" OR thiamine OR thiamines OR flavonoid OR flavonoids OR carotenoid OR carotenoids OR "fatty acid" OR "fatty acids" OR "omega-3" OR

"omega-6" OR "n-3" OR "n-6" or pufa OR lcpufa OR mufa OR "fish oil*" OR magnesium OR iodine OR "nutrient deficienc*" OR "nutritional supplement*" OR "dietary supplement*"))

#2
(TS = (child OR children OR childhood OR kid OR kids OR girl* OR boy* OR teenage* OR youth* OR youngster* OR preschool* OR pre-school* OR kindergarten* OR "elementary school" OR elementary-school OR juvenile* OR minors OR minor OR childhood OR pediatric* OR paediatric* OR schoolchild OR infant OR infants OR infancy OR toddler* OR teen OR teens))

#3
(TS = ("mental disorder*" OR "mental health disorder*" OR "mental illness*" OR "psychiatric disorder*" OR "affective disorder*" OR "psychopathology" OR "psychiatric diagnoses" OR "psychiatric diagnosis" **OR** "psychiatric illness*" OR "child development disorder*" OR "behavior disorder*" OR "behaviour disorder*" OR "externalizing disorder*" OR "internalizing disorder*"))

#4
(TS = (depression OR depressions OR depressive OR "bipolar disorder*" OR "unipolar disorder*" OR melancholia OR melancholias OR melancholic OR "manic-depressive disorder*" OR dysthymia or dysthymic OR cyclothymia OR cyclothymic OR "mood disorder*"))

#5
(TS = (Anxiety OR anxieties OR phobia OR phobias OR phobic OR "panic disorder*" OR "obsessive-compulsive disorder" OR "posttraumatic stress disorder" OR PTSD OR "post-traumatic stress disorder"))

#6
(TS = (dyslexi* OR dyscalcul* OR dysgraphia* OR agraphia* OR "academic skill disorder*" OR "academic skills disorder*" OR "scholastic skill disorder*" OR "learning difficult*" OR   "learning disorder*" OR "learning disabilit*" OR "learning deficien*" OR "learning problem*" OR "reading abilit*" OR "reading accuracy" OR "reading comprehension" OR "reading disorder*" OR "reading disabilit*" OR "reading difficult*" OR "reading deficien*" OR "reading fluency" OR "reading problem*" OR "reading speed*" OR "math difficult*" OR "mathematical difficult*" OR "math abilit*" OR "mathematical abilit*" OR "math accurac*" OR "mathematical accurac*" OR "math disorder*" OR "mathematical disorder" OR "math disabilit*" OR "mathematical disabilit*" OR "math problem*" OR "mathematical problem*" OR "math fluency" OR "spelling abilit*" OR "spelling accurac*" OR "spelling comprehension" OR "spelling disorder*" OR "spelling disabilit*" OR "spelling difficult*"   OR "spelling deficien*"   OR "spelling fluenc*"   OR "spelling problem*" OR "spelling speed" OR "writing abilit*" OR "writing accurac*" OR "writing comprehension" OR "writing disorder*" OR "writing disabilit*"   OR "writing difficult*"   OR "writing deficiency" OR "writing fluency" OR "writing problem*" OR "writing speed*"))

#7
(TS = ("attention deficit disorder" OR "attention deficit hyperactivity disorder" OR ADHD OR "hyperkinetic syndrome" OR hyperactiv* OR hyperkinesis* OR "Oppositional Defiant Disorder*"))

#8
(TS = ("pervasive development disorder*" OR "pervasive developmental disorder*" OR PDD OR PDDs OR ASD OR ASDs OR autis* OR Asperger* OR "autism spectrum disorder*" OR Kanner* OR Rett*))

#9
#1 AND #2

#10

#3 OR #4 OR #5 OR #6 OR #7 OR #8

#11

#9 AND #10

PsychInfo

TI “psychiatric illness” OR TI “severe mental disorder*” OR TI “mental disorder*” OR TI “mental health disorder*” OR TI “mental illness*” OR TI “psychiatric disorder*” OR TI “affective disorder*” OR TI psychopathology OR TI “psychiatric diagnoses” OR TI “psychiatric diagnosis” OR AB “psychiatric illness*” OR AB “severe mental disorder*” OR AB “mental disorder*” OR AB “mental health disorder*” OR AB “mental illness*” OR AB “psychiatric disorder*” OR AB “affective disorder*” OR AB psychopathology OR AB “psychiatric diagnoses” OR AB “psychiatric diagnosis”

OR

DE "Major Depression" OR DE "Anaclitic Depression" OR DE "Dysthymic Disorder" OR DE "Endogenous Depression" OR DE "Late Life Depression" OR DE "Postpartum Depression" OR DE "Reactive Depression" OR DE "Recurrent Depression" OR DE "Treatment Resistant Depression" OR TI depression OR TI Depressions OR TI depressive OR TI “bipolar disorder*” OR TI “unipolar disorder*” OR TI melancholia OR TI melancholias OR TI melancholic OR TI “manic-depressive disorder*” OR TI dysthymia OR TI dysthymic OR TI cyclothymia OR TI "cyclothymic" OR TI “mood disorder*” OR AB depression OR AB Depressions OR AB depressive OR AB “bipolar disorder*” OR AB “unipolar disorder*” OR AB melancholia OR AB melancholias OR AB melancholic OR AB “manic-depressive disorder*” OR AB dysthymia OR AB dysthymic OR AB cyclothymia OR AB "cyclothymic" OR AB “mood disorder*”

OR

DE "Anxiety Disorders" OR DE "Acute Stress Disorder" OR DE "Castration Anxiety" OR DE "Death Anxiety" OR DE "Generalized Anxiety Disorder" OR DE "Obsessive Compulsive Disorder" OR DE "Panic Disorder" OR DE "Phobias" OR DE "Post-Traumatic Stress" OR DE "Posttraumatic Stress Disorder" OR DE "Separation Anxiety Disorder" OR TI Anxiety OR TI Anxieties OR TI phobia OR TI phobias OR TI phobic OR TI “panic disorder*” OR TI “obsessive compulsive disorder*” OR TI “posttraumatic stress disorder*” OR TI "PTSD" OR TI “post-traumatic stress disorder*” OR AB Anxiety OR AB Anxieties OR AB phobia OR AB phobias OR AB phobic OR AB “panic disorder*” OR AB “obsessive compulsive disorder*” OR AB “posttraumatic stress disorder*” OR AB "PTSD" OR AB “post-traumatic stress disorder*”

OR

DE "Schizophrenia" OR TI Schizophrenia OR TI psychotic OR TI psychosis OR TI psychoses OR TI schizophrenic OR TI "At risk mental state" OR TI “delusional disorder*” OR TI “schizophreniform disorder*” OR TI “schizoaffective disorder*” OR AB Schizophrenia OR AB psychotic OR AB psychosis OR AB psychoses OR AB schizophrenic OR AB "At risk mental state" OR AB “delusional disorder*” OR AB “schizophreniform disorder*” OR AB “schizoaffective disorder*”

OR

TI “psychiatric illness” OR TI “severe mental disorder*” OR TI “mental disorder*” OR TI “mental health disorder*” OR TI “mental illness*” OR TI “psychiatric disorder*” OR TI “affective disorder*” OR TI psychopathology OR TI “psychiatric diagnoses” OR TI “psychiatric diagnosis” OR AB “psychiatric illness*” OR AB “severe mental disorder*” OR AB “mental disorder*” OR AB “mental

health disorder*” OR AB “mental illness*” OR AB “psychiatric disorder*” OR AB “affective disorder*” OR AB psychopathology OR AB “psychiatric diagnoses” OR AB “psychiatric diagnosis” OR DE "Major Depression" OR DE "Anaclitic Depression" OR DE "Dysthymic Disorder" OR DE "Endogenous Depression" OR DE "Late Life Depression" OR DE "Postpartum Depression" OR DE "Reactive Depression" OR DE "Recurrent Depression" OR DE "Treatment Resistant Depression" OR TI depression OR TI Depressions OR TI depressive OR TI “bipolar disorder*” OR TI “unipolar disorder*” OR TI melancholia OR TI melancholias OR TI melancholic OR TI “manic-depressive disorder*” OR TI dysthymia OR TI dysthymic OR TI cyclothymia OR TI "cyclothymic" OR TI “mood disorder*” OR AB depression OR AB Depressions OR AB depressive OR AB “bipolar disorder*” OR AB “unipolar disorder*” OR AB melancholia OR AB melancholias OR AB melancholic OR AB “manic-depressive disorder*” OR AB dysthymia OR AB dysthymic OR AB cyclothymia OR AB "cyclothymic" OR AB “mood disorder*” OR DE "Anxiety Disorders" OR DE "Acute Stress Disorder" OR DE "Castration Anxiety" OR DE "Death Anxiety" OR DE "Generalized Anxiety Disorder" OR DE "Obsessive Compulsive Disorder" OR DE "Panic Disorder" OR DE "Phobias" OR DE "Post-Traumatic Stress" OR DE "Posttraumatic Stress Disorder" OR DE "Separation Anxiety Disorder" OR TI Anxiety OR TI Anxieties OR TI phobia OR TI phobias OR TI phobic OR TI “panic disorder*” OR TI “obsessive compulsive disorder*” OR TI “posttraumatic stress disorder*” OR TI "PTSD" OR TI “post-traumatic stress disorder*” OR AB Anxiety OR AB Anxieties OR AB phobia OR AB phobias OR AB phobic OR AB “panic disorder*” OR AB “obsessive compulsive disorder*” OR AB “posttraumatic stress disorder*” OR AB "PTSD" OR AB “post-traumatic stress disorder*” OR DE "Schizophrenia" OR TI Schizophrenia OR TI psychotic OR TI psychosis OR TI psychoses OR TI schizophrenic OR TI "At risk mental state" OR TI “delusional disorder*” OR TI “schizophreniform disorder*” OR TI “schizoaffective disorder*” OR AB Schizophrenia OR AB psychotic OR AB psychosis OR AB psychoses OR AB schizophrenic OR AB "At risk mental state" OR AB “delusional disorder*” OR AB “schizophreniform disorder*” OR AB “schizoaffective disorder*”

OR

TI dyslexi* OR TI dyscalcul* OR TI dysgraphia* OR TI agraphia* OR TI “academic skill disorder*” OR TI “academic skills disorder*” OR TI “scholastic skill disorder*” OR TI “learning difficult*” OR TI “learning disorder*” OR TI “learning disabilit*” OR TI “learning deficien*” OR TI “learning problem*” OR TI “reading abilit*” OR TI “reading accuracy” OR TI “reading comprehension” OR TI “reading disorder*” OR TI “reading disabilit*” OR TI “reading difficult*” OR TI “reading deficien*” OR TI “reading fluency” OR TI “reading problem*” OR TI “reading speed*” OR TI “math difficult*” OR TI “mathematical difficult*” OR TI “math abilit*” OR TI “mathematical abilit*” OR TI “math accurac*” OR TI “mathematical accurac*” OR TI “math disorder*” OR TI “mathematical disorder*” OR TI “math disabilit*” OR TI “mathematical disabilit*” OR TI “math problem*” OR TI “mathematical problem*” OR TI “math fluency” OR TI “spelling abilit*” OR TI “spelling accurac*” OR TI “spelling comprehension” OR TI “spelling disorder*” OR TI “spelling disabilit*” OR TI “spelling difficult*” OR TI “spelling deficien*” OR TI “spelling fluenc*” OR TI “spelling problem*” OR TI “spelling speed” OR TI “writing abilit*” OR TI “writing accurac*” OR TI “writing comprehension” OR TI “writing disorder*” OR TI “writing disabilit*” OR TI “writing difficult*” OR TI "writing deficiency" OR TI “writing fluency” OR TI “writing problem*” OR TI “writing speed*” OR AB dyslexi* OR AB dyscalcul* OR AB dysgraphia* OR AB agraphia* OR AB “academic skill disorder*” OR AB “academic skills disorder*” OR AB “scholastic skill disorder*” OR AB “learning difficult*” OR AB “learning disorder*” OR AB “learning disabilit*” OR AB “learning deficien*” OR AB “learning problem*” OR AB “reading abilit*” OR AB “reading accuracy” OR AB “reading comprehension” OR AB “reading disorder*” OR AB “reading disabilit*” OR AB “reading difficult*” OR AB “reading deficien*” OR AB “reading fluency” OR AB “reading problem*” OR AB “reading speed*” OR AB “math difficult*” OR AB “mathematical difficult*” OR AB “math abilit*” OR AB “mathematical abilit*” OR AB “math accurac*” OR AB “mathematical accurac*” OR AB “math

disorder*” OR AB “mathematical disorder*” OR AB “math disabilit*” OR AB “mathematical disabilit*” OR AB “math problem*” OR AB “mathematical problem*” OR AB “math fluency” OR AB “spelling abilit*” OR AB “spelling accurac*” OR AB “spelling comprehension” OR AB “spelling disorder*” OR AB “spelling disabilit*” OR AB “spelling difficult*” OR AB “spelling deficien*” OR AB “spelling fluenc*” OR AB “spelling problem*” OR AB “spelling speed” OR AB “writing abilit*” OR AB “writing accurac*” OR AB “writing comprehension” OR AB “writing disorder*” OR AB “writing disabilit*” OR AB “writing difficult*” OR AB “writing deficiency” OR AB “writing fluency” OR AB “writing problem*” OR AB “writing speed*”

OR

TI “attention deficit disorder” OR TI “attention deficit hyperactivity disorder*” OR TI ADHD OR TI "hyperkinetic syndrome" OR TI hyperactiv* OR TI hyperkinesis OR TI "Oppositional Defiant Disorder*" OR AB “attention deficit disorder” OR AB “attention deficit hyperactivity disorder*” OR AB ADHD OR AB "hyperkinetic syndrome" OR AB hyperactiv* OR AB hyperkinesis OR AB "Oppositional Defiant Disorder*"

OR

TI “pervasive development disorder*” OR TI “pervasive developmental disorder*” OR TI PDD OR TI PDDs OR TI ASD OR TI ASDs OR TI autis* OR TI Asperger* OR TI “autism spectrum disorder*” OR TI Kanner* OR TI Rett* OR AB “pervasive development disorder*” OR AB “pervasive developmental disorder*” OR AB PDD OR AB PDDs OR AB ASD OR AB ASDs OR AB autis* OR AB Asperger* OR AB “autism spectrum disorder*” OR AB Kanner* OR AB Rett*

AND

TI “newborn infant” OR TI “newborn infants” OR TI child OR TI children OR TI childhood OR TI kid OR TI kids OR TI girl* OR TI boy* OR TI teenage* OR TI youth* OR TI youngster* OR TI preschool* OR TI pre-school* OR TI kindergarten* OR TI “elementary school” OR TI elementary-school OR TI juvenile* OR TI minors OR TI minor OR TI pediatric* OR TI paediatric* OR TI schoolchild* OR TI infant OR TI infants OR TI infancy OR TI toddler* OR TI teen OR TI teens OR AB child OR AB children OR AB childhood OR AB kid OR AB kids OR AB girl* OR AB boy* OR AB teenage* OR AB youth* OR AB youngster* OR AB preschool* OR AB pre-school* OR AB kindergarten* OR AB “elementary school” OR AB elementary-school OR AB juvenile* OR AB minors OR AB minor OR AB pediatric* OR AB paediatric* OR AB schoolchild* OR AB infant OR AB infants OR AB infancy OR AB toddler* OR AB teen OR AB teens OR AB “newborn infant” OR AB “newborn infants”

AND

DE "Diets" OR DE "Food" OR TI “dietary pattern*” OR TI “dietary intake*” OR TI “food intake*” OR TI “food pattern*” OR TI “low energy diet*” OR TI “high energy diet*” OR TI “healthy diet*” OR TI “prudent diet*” OR TI “mediterranean diet*” OR TI “western diet*” OR TI “traditional diet*” OR TI “fast food*” OR TI “junk food*” OR TI snack OR TI snacks OR TI nut OR TI nuts OR TI fruit OR TI fruits OR TI vegetable OR TI vegetables OR TI legume OR TI legumes OR TI “vegetarian diet*” OR TI “vegan diet*” OR TI "diet quality" OR TI "dietary quality" OR TI “glycaemic index*” OR TI “mind diet*” OR TI “nordic diet*” OR TI “calorie restriction*” OR TI “caloric restriction*” OR TI "high caloric" OR TI “high calorie*” OR TI nutrition OR AB “dietary pattern*” OR AB “dietary intake*” OR AB “food intake*” OR AB “food pattern*” OR AB “low energy diet*” OR AB “high energy diet*” OR AB “healthy diet*” OR AB “prudent diet*” OR AB “mediterranean diet*” OR AB “western diet*” OR AB “traditional diet*” OR AB “fast food*” OR AB “junk food*” OR AB snack OR AB snacks OR AB nut OR AB nuts OR AB fruit OR AB fruits OR AB vegetable OR AB vegetables OR AB legume OR AB legumes OR AB “vegetarian diet*” OR AB “vegan diet*” OR AB

"diet quality" OR AB "dietary quality" OR AB “glycaemic index*” OR AB “mind diet*” OR AB “nordic diet*” OR AB “calorie restriction*” OR AB “caloric restriction*” OR AB "high caloric" OR AB “high calorie*” OR AB nutrition

OR

TI vitamin OR TI zinc OR TI iron OR TI selenium OR TI copper OR TI gluten OR TI folate OR TI folates OR TI “folic acid*” OR TI thiamine OR TI thiamines OR TI flavonoid OR TI flavonoids OR TI carotenoid OR TI carotenoids OR TI "fatty acid" OR TI "fatty acids" OR TI "omega-3" OR TI "omega-6" OR TI "n-3" OR TI "n-6" OR TI pufa OR TI lcpufa OR TI mufa OR TI “fish oil*” OR TI magnesium OR TI iodine OR TI “nutrient deficienc*” OR TI “nutritional supplement*” OR TI “dietary supplement*” OR AB vitamin OR AB zinc OR AB iron OR AB selenium OR AB copper OR AB gluten OR AB folate OR AB folates OR AB “folic acid*” OR AB thiamine OR AB thiamines OR AB flavonoid OR AB flavonoids OR AB carotenoid OR AB carotenoids OR AB "fatty acid" OR AB "fatty acids" OR AB "omega-3" OR AB "omega-6" OR AB "n-3" OR AB "n-6" OR AB pufa OR AB lcpufa OR AB mufa OR AB “fish oil*” OR AB magnesium OR AB iodine OR AB “nutrient deficienc*” OR AB “nutritional supplement*” OR AB “dietary supplement*”

Cochrane

#1 [mh diet] OR [mh food] OR "dietary pattern*":ti,ab OR "dietary intake*":ti,ab OR "food intake*":ti,ab OR "food pattern*":ti,ab OR " low energy diet*":ti,ab OR " high energy diet*":ti,ab OR "healthy diet*":ti,ab OR " prudent diet*":ti,ab OR "mediterranean diet*":ti,ab OR "western diet*":ti,ab OR "traditional diet*":ti,ab OR "fast food*":ti,ab OR "junk food*":ti,ab OR snack:ti,ab OR snacks:ti,ab OR nut:ti,ab OR nuts:ti,ab OR fruit:ti,ab OR fruits:ti,ab OR vegetable:ti,ab OR vegetables:ti,ab OR legume:ti,ab OR legumes:ti,ab OR "vegetarian diet*":ti,ab OR "vegan diet*":ti,ab OR "diet quality":ti,ab OR "dietary quality":ti,ab OR "glycaemic index*":ti,ab OR "mind diet*":ti,ab OR "nordic diet*":ti,ab OR "calorie restriction*":ti,ab OR "caloric restriction*":ti,ab OR "high caloric":ti,ab OR "high calorie*":ti,ab OR nutrition:ti,ab

OR

vitamin:ti,ab OR zinc:ti,ab OR iron:ti,ab OR selenium:ti,ab OR copper:ti,ab OR gluten:ti,ab OR folate:ti,ab OR folates:ti,ab OR "folic acid*":ti,ab OR thiamine:ti,ab OR thiamines:ti,ab OR flavonoid:ti,ab OR flavonoids:ti,ab OR carotenoid:ti,ab OR carotenoids:ti,ab OR "fatty acid":ti,ab OR "fatty acids":ti,ab OR "omega-3":ti,ab OR "omega-6":ti,ab OR "n-3":ti,ab OR "n-6":ti,ab OR pufa:ti,ab OR lcpufa:ti,ab OR mufa:ti,ab OR "fish oil*":ti,ab OR magnesium:ti,ab OR iodine:ti,ab OR "nutrient deficienc*":ti,ab OR " nutritional supplement*":ti,ab OR "dietary supplement*":ti,ab

#2 "mental disorder*":ti,ab OR "mental health disorder*":ti,ab OR "mental illness*":ti,ab OR "psychiatric disorder*":ti,ab OR "affective disorder*":ti,ab OR psychopathology:ti,ab OR "psychiatric diagnoses":ti,ab OR "psychiatric diagnosis":ti,ab OR "psychiatric illness*":ti,ab OR "child development disorder*":ti,ab OR "behavior disorder*":ti,ab OR "behaviour disorder*":ti,ab OR "externalizing disorder*":ti,ab OR "internalizing disorder*":ti,ab [mh "neurodevelopmental disorders"]

OR

[mh depression] OR depression:ti,ab OR depressions:ti,ab OR depressive:ti,ab OR "bipolar disorder*":ti,ab OR "unipolar disorder*":ti,ab OR melancholia:ti,ab OR melancholias:ti,ab OR melancholic:ti,ab OR "manic-depressive disorder*":ti,ab OR dysthymia:ti,ab OR dysthymic:ti,ab OR cyclothymia:ti,ab OR cyclothymic:ti,ab OR [mh "mood disorders"] OR "mood disorder*":ti,ab

OR

[mh "anxiety disorders"] OR anxiety:ti,ab OR anxieties:ti,ab OR phobia:ti,ab OR phobias:ti,ab OR phobic:ti,ab OR "panic disorder*":ti,ab OR "obsessive compulsive disorder*":ti,ab OR "posttraumatic stress disorder*":ti,ab OR PTSD:ti,ab OR "post-traumatic stress disorder*":ti,ab OR [mh "stress disorders, posttraumatic"]

OR

dyslexi*:ti,ab OR dyscalcul*:ti,ab OR dysgraphia*:ti,ab OR agraphia*:ti,ab OR "academic skill disorder*":ti,ab OR "scholastic skill disorder*":ti,ab OR "learning difficult*":ti,ab OR "learning disorder*":ti,ab OR "learning disabilit*":ti,ab OR "learning deficien*":ti,ab OR "learning problem*":ti,ab OR "reading abilit*":ti,ab OR "reading accuracy":ti,ab OR "reading comprehension":ti,ab OR "reading disorder*":ti,ab OR "reading disabilit*":ti,ab OR "reading difficult*":ti,ab OR "reading deficien*":ti,ab OR "reading fluency":ti,ab OR "reading problem*":ti,ab OR "reading speed*":ti,ab OR "math difficult*":ti,ab OR "mathematical difficult*":ti,ab OR "mathematical abilit*":ti,ab OR "math abilit*":ti,ab OR "math accurac*":ti,ab OR "mathematical accurac*":ti,ab OR "math disorder*":ti,ab OR "mathematical disorder*":ti,ab OR "math disabilit*":ti,ab OR "mathematical disabilit*":ti,ab OR "math problem*":ti,ab OR "mathematical problem*":ti,ab OR "math fluency ":ti,ab OR "spelling abilit*":ti,ab OR "spelling accurac*":ti,ab OR "spelling comprehension ":ti,ab OR "spelling disorder*":ti,ab OR "spelling disabilit*":ti,ab OR "spelling difficult*":ti,ab OR "spelling deficien*":ti,ab OR "spelling fluenc*":ti,ab OR "spelling problem*":ti,ab OR "spelling speed ":ti,ab OR "writing abilit*":ti,ab OR "writing accurac*":ti,ab OR "writing comprehension":ti,ab OR "writing disorder*":ti,ab OR "writing disabilit*":ti,ab OR "writing difficult*":ti,ab OR "writing deficiency":ti,ab OR "writing fluency":ti,ab OR "writing problem*":ti,ab OR "writing speed*":ti,ab OR [mh Agraphia]

OR

"attention deficit disorder*":ti,ab OR "attention deficit hyperactivity disorder*":ti,ab OR ADHD:ti,ab OR "hyperkinetic syndrome":ti,ab OR hyperactiv*:ti,ab OR hyperkinesis:ti,ab OR "Oppositional Defiant Disorder*":ti,ab

OR

"pervasive development disorder*":ti,ab OR "pervasive developmental disorder*":ti,ab OR PDD:ti,ab OR PDDs:ti,ab OR ASD:ti,ab OR ASDs:ti,ab OR autis*:ti,ab OR Asperger*:ti,ab OR "autism spectrum disorder*":ti,ab OR Kanner*:ti,ab OR Rett*:ti,ab

#3

child:ti,ab OR children:ti,ab OR childhood:ti,ab OR kid:ti,ab OR kids:ti,ab OR girl*:ti,ab OR boy*:ti,ab OR teenage*:ti,ab OR youth*:ti,ab OR youngster*:ti,ab OR preschool*:ti,ab OR pre-school*:ti,ab OR kindergarten*:ti,ab OR "elementary school":ti,ab OR "elementary-school":ti,ab OR juvenile*:ti,ab OR minors:ti,ab OR minor:ti,ab OR pediatric*:ti,ab OR paediatric*:ti,ab OR schoolchild*:ti,ab OR infant:ti,ab OR infants:ti,ab OR infancy:ti,ab OR toddler*:ti,ab OR teen:ti,ab OR teens:ti,ab OR [mh "child, preschool"] OR [mh child] OR [mh infant] OR [mh "infant, newborn"]

#1 AND #2 AND #3

CINAHL

TI “psychiatric illness” OR TI “severe mental disorder*” OR TI “mental disorder*” OR TI “mental health disorder*” OR TI “mental illness*” OR TI “psychiatric disorder*” OR TI “affective disorder*” OR TI psychopathology OR TI “psychiatric diagnoses” OR TI “psychiatric diagnosis” OR AB “psychiatric illness*” OR AB “severe mental disorder*” OR AB “mental disorder*” OR AB “mental health disorder*” OR AB “mental illness*” OR AB “psychiatric disorder*” OR AB “affective disorder*” OR AB psychopathology OR AB “psychiatric diagnoses” OR AB “psychiatric diagnosis”

OR

(MH "Depression+") OR TI depression OR TI Depressions OR TI depressive OR TI “bipolar disorder*” OR TI “unipolar disorder*” OR TI melancholia OR TI melancholias OR TI melancholic OR TI “manic-depressive disorder*” OR TI dysthymia OR TI dysthymic OR TI cyclothymia OR TI "cyclothymic" OR TI “mood disorder*” OR AB depression OR AB Depressions OR AB depressive OR AB “bipolar disorder*” OR AB “unipolar disorder*” OR AB melancholia OR AB melancholias OR AB melancholic OR AB “manic-depressive disorder*” OR AB dysthymia OR AB dysthymic OR AB cyclothymia OR AB "cyclothymic" OR AB “mood disorder*”

OR

(MH "Anxiety Disorders+") OR TI Anxiety OR TI Anxieties OR TI phobia OR TI phobias OR TI phobic OR TI “panic disorder*” OR TI “obsessive compulsive disorder*” OR TI “posttraumatic stress disorder*” OR TI "PTSD" OR TI “post-traumatic stress disorder*” OR AB Anxiety OR AB Anxieties OR AB phobia OR AB phobias OR AB phobic OR AB “panic disorder*” OR AB “obsessive compulsive disorder*” OR AB “posttraumatic stress disorder*” OR AB "PTSD" OR AB “post-traumatic stress disorder*”

OR

(MH "Psychotic Disorders+") OR (MH "Schizophrenia+") OR TI Schizophrenia OR TI psychotic OR TI psychosis OR TI psychoses OR TI schizophrenic OR TI "At risk mental state" OR TI “delusional disorder*” OR TI “schizophreniform disorder*” OR TI “schizoaffective disorder*” OR AB Schizophrenia OR AB psychotic OR AB psychosis OR AB psychoses OR AB schizophrenic OR AB "At risk mental state" OR AB “delusional disorder*” OR AB “schizophreniform disorder*” OR AB “schizoaffective disorder*”

OR

TI “psychiatric illness” OR TI “severe mental disorder*” OR TI “mental disorder*” OR TI “mental health disorder*” OR TI “mental illness*” OR TI “psychiatric disorder*” OR TI “affective disorder*” OR TI psychopathology OR TI “psychiatric diagnoses” OR TI “psychiatric diagnosis” OR AB “psychiatric illness*” OR AB “severe mental disorder*” OR AB “mental disorder*” OR AB “mental health disorder*” OR AB “mental illness*” OR AB “psychiatric disorder*” OR AB “affective disorder*” OR AB psychopathology OR AB “psychiatric diagnoses” OR AB “psychiatric diagnosis” OR (MH "Depression+") OR TI depression OR TI Depressions OR TI depressive OR TI “bipolar disorder*” OR TI “unipolar disorder*” OR TI melancholia OR TI melancholias OR TI melancholic OR TI “manic-depressive disorder*” OR TI dysthymia OR TI dysthymic OR TI cyclothymia OR TI "cyclothymic" OR TI “mood disorder*” OR AB depression OR AB Depressions OR AB depressive OR AB “bipolar disorder*” OR AB “unipolar disorder*” OR AB melancholia OR AB melancholias OR AB melancholic OR AB “manic-depressive disorder*” OR AB dysthymia OR AB dysthymic OR AB cyclothymia OR AB "cyclothymic" OR AB “mood disorder*” OR (MH "Anxiety Disorders+") OR TI Anxiety OR TI Anxieties OR TI phobia OR TI phobias OR TI phobic OR TI “panic disorder*” OR TI “obsessive compulsive disorder*” OR TI “posttraumatic stress disorder*” OR TI "PTSD" OR TI “post-traumatic stress disorder*” OR AB Anxiety OR AB Anxieties OR AB phobia OR AB

phobias OR AB phobic OR AB “panic disorder*” OR AB “obsessive compulsive disorder*” OR AB “posttraumatic stress disorder*” OR AB "PTSD" OR AB “post-traumatic stress disorder*” OR (MH "Psychotic Disorders+") OR (MH "Schizophrenia+") OR TI Schizophrenia OR TI psychotic OR TI psychosis OR TI psychoses OR TI schizophrenic OR TI "At risk mental state" OR TI “delusional disorder*” OR TI “schizophreniform disorder*” OR TI “schizoaffective disorder*” OR AB Schizophrenia OR AB psychotic OR AB psychosis OR AB psychoses OR AB schizophrenic OR AB "At risk mental state" OR AB “delusional disorder*” OR AB “schizophreniform disorder*” OR AB “schizoaffective disorder*”

OR

TI dyslexi* OR TI dyscalcul* OR TI dysgraphia* OR TI agraphia* OR TI “academic skill disorder*” OR TI “academic skills disorder*” OR TI “scholastic skill disorder*” OR TI “learning difficult*” OR TI “learning disorder*” OR TI “learning disabilit*” OR TI “learning deficien*” OR TI “learning problem*” OR TI “reading abilit*” OR TI “reading accuracy” OR TI “reading comprehension” OR TI “reading disorder*” OR TI “reading disabilit*” OR TI “reading difficult*” OR TI “reading deficien*” OR TI “reading fluency” OR TI “reading problem*” OR TI “reading speed*” OR TI “math difficult*” OR TI “mathematical difficult*” OR TI “math abilit*” OR TI “mathematical abilit*” OR TI “math accurac*” OR TI “mathematical accurac*” OR TI “math disorder*” OR TI “mathematical disorder*” OR TI “math disabilit*” OR TI “mathematical disabilit*” OR TI “math problem*” OR TI “mathematical problem*” OR TI “math fluency” OR TI “spelling abilit*” OR TI “spelling accurac*” OR TI “spelling comprehension” OR TI “spelling disorder*” OR TI “spelling disabilit*” OR TI “spelling difficult*” OR TI “spelling deficien*” OR TI “spelling fluenc*” OR TI “spelling problem*” OR TI “spelling speed” OR TI “writing abilit*” OR TI “writing accurac*” OR TI “writing comprehension” OR TI “writing disorder*” OR TI “writing disabilit*” OR TI “writing difficult*” OR TI "writing deficiency" OR TI “writing fluency” OR TI “writing problem*” OR TI “writing speed*” OR (MH "Agraphia+") OR AB dyslexi* OR AB dyscalcul* OR AB dysgraphia* OR AB agraphia* OR AB “academic skill disorder*” OR AB “academic skills disorder*” OR AB “scholastic skill disorder*” OR AB “learning difficult*” OR AB “learning disorder*” OR AB “learning disabilit*” OR AB “learning deficien*” OR AB “learning problem*” OR AB “reading abilit*” OR AB “reading accuracy” OR AB “reading comprehension” OR AB “reading disorder*” OR AB “reading disabilit*” OR AB “reading difficult*” OR AB “reading deficien*” OR AB “reading fluency” OR AB “reading problem*” OR AB “reading speed*” OR AB “math difficult*” OR AB “mathematical difficult*” OR AB “math abilit*” OR AB “mathematical abilit*” OR AB “math accurac*” OR AB “mathematical accurac*” OR AB “math disorder*” OR AB “mathematical disorder*” OR AB “math disabilit*” OR AB “mathematical disabilit*” OR AB “math problem*” OR AB “mathematical problem*” OR AB “math fluency” OR AB “spelling abilit*” OR AB “spelling accurac*” OR AB “spelling comprehension” OR AB “spelling disorder*” OR AB “spelling disabilit*” OR AB “spelling difficult*” OR AB “spelling deficien*” OR AB “spelling fluenc*” OR AB “spelling problem*” OR AB “spelling speed” OR AB “writing abilit*” OR AB “writing accurac*” OR AB “writing comprehension” OR AB “writing disorder*” OR AB “writing disabilit*” OR AB “writing difficult*” OR AB “writing deficiency” OR AB “writing fluency” OR AB “writing problem*” OR AB “writing speed*”

OR

TI “attention deficit disorder” OR TI “attention deficit hyperactivity disorder*” OR TI ADHD OR TI "hyperkinetic syndrome" OR TI hyperactiv* OR TI hyperkinesis OR TI "Oppositional Defiant Disorder*" OR AB “attention deficit disorder” OR AB “attention deficit hyperactivity disorder*” OR AB ADHD OR AB "hyperkinetic syndrome" OR AB hyperactiv* OR AB hyperkinesis OR AB "Oppositional Defiant Disorder*"

OR

TI “pervasive development disorder*” OR TI “pervasive developmental disorder*” OR TI PDD OR TI PDDs OR TI ASD OR TI ASDs OR TI autis* OR TI Asperger* OR TI “autism spectrum disorder*” OR TI Kanner* OR TI Rett* OR AB “pervasive development disorder*” OR AB “pervasive developmental disorder*” OR AB PDD OR AB PDDs OR AB ASD OR AB ASDs OR AB autis* OR AB Asperger* OR AB “autism spectrum disorder*” OR AB Kanner* OR AB Rett*

AND

(MH "Child, Preschool") OR (MH "Child+") OR (MH "Infant+") OR (MH "Infant, Newborn+") OR TI child OR TI children OR TI childhood OR TI kid OR TI kids OR TI girl* OR TI boy* OR TI teenage* OR TI youth* OR TI youngster* OR TI preschool* OR TI pre-school* OR TI kindergarten* OR TI “elementary school” OR TI elementary-school OR TI juvenile* OR TI minors OR TI minor OR TI pediatric* OR TI paediatric* OR TI schoolchild* OR TI infant OR TI infants OR TI infancy OR TI toddler* OR TI teen OR TI teens OR AB child OR AB children OR AB childhood OR AB kid OR AB kids OR AB girl* OR AB boy* OR AB teenage* OR AB youth* OR AB youngster* OR AB preschool* OR AB pre-school* OR AB kindergarten* OR AB “elementary school” OR AB elementary-school OR AB juvenile* OR AB minors OR AB minor OR AB pediatric* OR AB paediatric* OR AB schoolchild* OR AB infant OR AB infants OR AB infancy OR AB toddler* OR AB teen OR AB teens

AND

(MH "Diet+") OR (MH "Food+") OR TI “dietary pattern*” OR TI “dietary intake*” OR TI “food intake*” OR TI “food pattern*” OR TI “low energy diet*” OR TI “high energy diet*” OR TI “healthy diet*” OR TI “prudent diet*” OR TI “mediterranean diet*” OR TI “western diet*” OR TI “traditional diet*” OR TI “fast food*” OR TI “junk food*” OR TI snack OR TI snacks OR TI nut OR TI nuts OR TI fruit OR TI fruits OR TI vegetable OR TI vegetables OR TI legume OR TI legumes OR TI “vegetarian diet*” OR TI “vegan diet*” OR TI "diet quality" OR TI "dietary quality" OR TI “glycaemic index*” OR TI “mind diet*” OR TI “nordic diet*” OR TI “calorie restriction*” OR TI “caloric restriction*” OR TI "high caloric" OR TI “high calorie*” OR TI nutrition OR AB “dietary pattern*” OR AB “dietary intake*” OR AB “food intake*” OR AB “food pattern*” OR AB “low energy diet*” OR AB “high energy diet*” OR AB “healthy diet*” OR AB “prudent diet*” OR AB “mediterranean diet*” OR AB “western diet*” OR AB “traditional diet*” OR AB “fast food*” OR AB “junk food*” OR AB snack OR AB snacks OR AB nut OR AB nuts OR AB fruit OR AB fruits OR AB vegetable OR AB vegetables OR AB legume OR AB legumes OR AB “vegetarian diet*” OR AB “vegan diet*” OR AB "diet quality" OR AB "dietary quality" OR AB “glycaemic index*” OR AB “mind diet*” OR AB “nordic diet*” OR AB “calorie restriction*” OR AB “caloric restriction*” OR AB "high caloric" OR AB “high calorie*” OR AB nutrition

OR

TI vitamin OR TI zinc OR TI iron OR TI selenium OR TI copper OR TI gluten OR TI folate OR TI folates OR TI “folic acid*”OR TI thiamine OR TI thiamines OR TI flavonoid OR TI flavonoids OR TI carotenoid OR TI carotenoids OR TI "fatty acid" OR TI "fatty acids" OR TI "omega-3" OR TI "omega-6" OR TI "n-3" OR TI "n-6" OR TI pufa OR TI lcpufa OR TI mufa OR TI “fish oil*” OR TI magnesium OR TI iodine OR TI “nutrient deficienc*” OR TI “nutritional supplement*” OR TI “dietary supplement*” OR AB vitamin OR AB zinc OR AB iron OR AB selenium OR AB copper OR AB gluten OR AB folate OR AB folates OR AB “folic acid*”OR AB thiamine OR AB thiamines OR AB flavonoid OR AB flavonoids OR AB carotenoid OR AB carotenoids OR AB "fatty acid" OR AB "fatty acids" OR AB "omega-3" OR AB "omega-6" OR AB "n-3" OR AB "n-6" OR AB pufa OR AB lcpufa OR AB mufa OR AB “fish oil*” OR AB magnesium OR AB iodine OR AB “nutrient deficienc*” OR AB “nutritional supplement*” OR AB “dietary supplement*”
