# Supplementary material for: A Scoping Literature Review of the Relation between Nutrition and ASD Symptoms in Children
Source: Nutrients. 2022 Mar 26;14(7):1389. doi: 10.3390/nu14071389 (PMC9003544; doi:10.3390/nu14071389)
Supplement: Supplementary file 1 [file nutrients-14-01389-s001.zip › File S2_included_articles_revised.pdf]

Supplementary File S2: Original articles in systematic reviews and meta-analyses.

Original articles in each systematic review/meta-analysis. The systematic review/meta-analysis is noted in bold, the non-bold articles below the bold name are the original articles included in the systematic review/meta-analyses and utilized in the current review.

Agostoni 2017 [1]
Bent 2014
Voigt 2014

Bent 2009 [2]
Amminger 2007
Bell 2004
Meguid 2008

Bostock 2017 [3]
Evangeliou 2003

Brondino 2015 [4]
Adams 2011
Al-Ayadhi 2013
Amminger 2007
Bashir 2014
Bent 2011
Bent 2014
Bertoglio 2010
Chan 2012
Chez 2002
Dolske 1993
Elder 2006
Evangeliou 2003
Findling 1997
Frye 2013
Johnson 2011
Knivsberg 2002
Meguid 2008
Taliou 2013
Tolbert 1993
Voigt 2014
Whiteley 2010

Buie 2013 [5]
Elder 2006
Knibsberg 2002
Knivsberg 1990
Lucardelli 1995
Sponheim 1991
Whiteley 1999

Castro 2015 [6]
Evangeliou 2003
Spilioti 2013

Cheng 2017 [7]
Amminger 2007
Bent 2011

Bent 2014
Mankad 2015
Voigt 2014

Christison 2006 [8]
Cade 2000
Knivsberg 1990
Knivsberg 1995
Lucardeli 1995
Reichelt 1990
Whiteley 1999

De Crescenzo 2020 [9]
Amminger 2007
Bent 2011
Bent 2014
Johnson 2010
Mankad 2015
Mazahery 2019
Parellada 2017
Voigt 2014

Focker 2017 [10]
Azzam 2015
Feng 2016
Saad 2016 (retracted)
Ucuz 2015

Fraguas 2019 [11]
Adams 2004
Adams 2011
Amminger 2007
Bent 2011
Bent 2014
Bertoglio 2010
Dolske 1993
Findling 1997
Frye 2016
Mankand 2015
Parellada 2017
Rimland 1978
Voigt 2014

Gillberg 2017 [12]
Azzam 2015
Saad 2016 (retracted)
Ucuz 2015

Gogou 2017 [13]
Amminger 2007
Bent 2011
Bent 2014
Bertoglio 2010
Dolske 1993
Elder 2006
Evangliou 2003

Findling 1997
Ghanizadeh 2013
Hardan 2012
Hyman 2016
Kern 2001
Knivsberg 2002
Lee 2018
Levine 1997
Martineau 1985
Minshavi 2016
Nikoo 2015
Posey 2004
Tolbert 1993
Voigt 2014
Whiteley 2010
Wink 2016

Gogou 2018 [14]
Elder 2006
Evangliou 2003
Hyman 2016
Knivsberg 2002
Lee 2018
Whiteley 2010

Horvath 2017 [15]
Amminger 2007
Bent 2011
Bent 2014
Mankad 2015
Voigt 2014

Hurwitz 2013 [16]
Elder 2006
Johnson 2011
Knivsberg 2003
Whiteley 2010

James 2011 [17]
Aminger 2007
Bent 2011

Kleijnen 1991 [18]
Barthelemy 1980
Jonas 1984
Martineau 1985
Rimland 1978

Kraeuter 2020 [19]
El-Rashidy 2017
Evangeliou 2003
Lee 2018

Li 2017a [20]
Al-Ayadhi 2013
Amminger 2007

Bashir 2014
Bent 2011
Bent 2014
Bertoglio 2010
Elder 2006
Frye 2016
Hendren 2016
Hyman 2016
Johnson 2011
Knivsberg 2003
Mankad 2015
Saad 2016 (retracted)
Voigt 2014
Whiteley 2010

Li 2017b [21]
Findling 1997
Frye 2016
Hendren 2016
Kerley 2017
Lelord 1981
Martineau 1985
Saad 2016 (retracted)
Tolbert 1993

Malaguarnera 2019 [22]
Fahmy 2013
Geier 2011
Goin-Kochel 2019

Mari-Bauset 2014 [23]
Cade 2000
Elder 2006
Goodwin 1971
Knivsberg 1990
Knivsberg 1995
Knivsberg 2002
Lucardelli 1995
Patel 2007
Reichelt 1990
Seung 2007
Whiteley 1999
Whiteley 2010

Mazahery 2016 [24]
Azzam 2015
Feng 2016
Saad 2016 (retracted)
Ucuz 2015

Millward 2008 + 2004 [25, 26]
Elder 2006
Knivsberg 2002/2003

Monteiro 2020 [27]
Adams 2004
Al-Ayadhi 2013

Amminger 2007
Bent 2011
Bent 2014
Chan 2012
El-Rashidy 2017
Elder 2006
Ghalichi 2016
Guo 2018
Hendren 2016
Hyman 2016
Johnson 2011
Knivsberg 2002
Navarro 2015
Pusponegoro 2015
Voigt 2014
Whiteley 2010

Mulloy 2010 + 2011 [28, 29]
Cade 2000
Elder 2006
Knivsberg 2002
Knivsberg 1990
Knivsberg 1995
Lucardelli 1995
Patel 2007
Reichelt 1990
Seung 2007
Whiteley 1999
Whiteley 2010

Murza 2010 [30]
Adams 2006

Nye 2002 [31]
Findling 1997
Tolbert 1993

Piwowarczyk 2018 [32]
Elder 2006
Jonhson 2011
Knivsberg 2002
Navarro 2015
Pusponegoro 2015
Whiteley 2010

Roux 2015 [33]
Amminger-2007
Bent 2011
Bent 2014
Johnson 2010
Mankad 2015
Meiri 2009

Sathe 2017 [34]
Al-Ayadi 2013
Bent 2011

Bent 2014
Bertoglio 2010
Elder 2006
Fahmy 2013
Geier 2011
Hendren 2016
Johnson 2010
Knivsberg 2002
Knivsberg 2003
Mankad 2015
Pedersen 2014
Seung 2007
Voigt 2014
Whiteley 2010

Williamson 2017 [35]
Al-Ayadhi 2013
Bent 2011
Bent 2014
Bertoglio 2010
Elder 2006
Fahmy 2013
Geier 2011
Ghalichi 2016
Hendren 2016
Johnson 2011
Knivsberg 2002
Knivsberg 2003
Mankad 2015
Navarro 2015
Pedersen 2014
Seung 2007
Voigt 2014
Whiteley 2010

Original articles in each in systematic review/meta-analyses per topic

The topic is noted in bold, the author and year in the first column refers to the original articles included in the systematic review/meta-analyses and utilized in the current umbrella review. In the second column it is noted in what meta-analys(is)/(es)/ and or systematic review(s) (name first author and year) the article was present.

**GFCF diet**	
Cade 2000	Christison 2006, Mari-Bauset 2014, Mulloy 2010 + 2011
Elder 2006	Brondino 2015, Buie 2013, Gogou 2017, Gogou 2018, Hurwitz 2013, Li 2017a, Mari-Bauset 2014, Millward 2008 + 2004, Monteiro 2020, Mulloy 2010 + 2011, Piwowarczyk 2018, Sathe 2017,Williamson 2017
Ghalichi 2016	Monteiro 2020, Williamson 2017
Goodwin 1971	Mari-Bauset 2014
Hyman 2016	Gogou 2017, Gogou 2018, Li 2017a
Johnson 2011	Brondino 2015, Hurwitz 2013, Li 2017a, Monteiro 2020, Williamson 2017
Knivsberg 2002	Brondino 2015, Gogou 2017, Gogou 2018, Mari-Bauset 2014, Millward 2008 + 2004, Monteiro 2020, Mulloy 2010 + 2011, Piwowarczyk 2018, Sathe 2017, Williamson 2017
Knivsberg 2003	Hurwitz 2013, Li 2017a, Millward 2008 + 2004, Sathe 2017, Williamson 2017
Knivsberg 1990	Buie 2013, Christison 2006, Mari-Bauset 2014, Mulloy 2010 + 2011
Knivsberg 1995 (same study as Knivsberg 1990)	Christison 2006, Mari-Bauset 2014, Mulloy 2010 + 2011
Lucarelli 1995	Buie 2013, Mari-Bauset 2014, Mulloy 2010 + 2011
Navarro 2015	Monteiro 2020, Piwowarczyk 2018, Williamson 2017
Patel 2007	Mari-Bauset 2014, Mulloy 2010 + 2011
Pedersen 2014 (same study as Whiteley 2010)	Sathe 2017, Williamson 2017
Pusponegoro 2015	Monteiro 2020, Piwowarczyk 2018
Reichelt 1990	Christison 2006, Mari-Bauset 2014, Mulloy 2010 + 2011
Seung 2007	Mari-Bauset 2014, Mulloy 2010 + 2011, Sathe 2017, Williamson 2017
Sponheim 1991	Buie 2013
Whiteley 1999	Buie 2013, Christison 2006, Mari-Bauset 2014, Mulloy 2010 + 2011
Whiteley 2010	Brondino 2015, Gogou 2017, Gogou 2018, Gogou 2018, Li 2017a, Hurwitz 2013, Mari-Bauset 2014, Monteiro 2020, Mulloy 2010 + 2011, Piwowarczyk 2018, Sathe 2017, Williamson 2017
**GFCF and ketogenic diet**	
El-Rashidy 2017	Kraeuter 2020, Monteiro 2020
**Ketogenic diet**	
Evangeliou 2003	Bostock 2017, Brondino 2015, Castro 2015, Kraeuter 2020
Lee 2018	Gogou 2017, Gogou 2018, Kraeuter 2020
Spilioti 2013	Castro 2015
**Chanyi diet**	
Chan 2012	Brondino 2015, Monteiro 2020
**Camel milk**	
Al-Ayadhi 2013	Brondino 2015, Li 2017a, Monteiro 2020, Williamson 2017
Bashir 2014	Brondino 2015, Li 2017a
**Omega-3 fatty acid**	
Amminger 2007	Bent 2009, Brondino 2015, Cheng 2017, De Crescenzo 2020, Fraguas 2019, Gogou 2017, Horvath 2017, Li 2017a, Monteiro 2020, Roux 2015
Bell 2004	Bent 2009

Bent 2011	Brondino 2015, Cheng 2017, De Crescenzo 2020, Fraguas 2019, Gogou 2017, Horvath 2017, James 2011, Li 2017a, Monteiro 2020, Roux 2015, Sathe 2017, Williamson 2017
Bent 2014	Brondino 2015, Cheng 2017, De Crescenzo 2020, Fraguas 2019, Gogou 2017, Horvath 2017, Li 2017a, Monteiro 2020, Roux 2015, Sathe 2017, Williamson 2017
Johnson 2010	De Crescenzo 2020, Roux 2015, Sathe 2017
Mankad 2015	Cheng 2017, De Crescenzo 2020, Horvath 2017, James 2011, Li 2017a, Roux 2015, Sathe 2017, Williamson 2017
Mazahery 2019	De Crescenzo 2020
Meguid 2008	Bent 2009, Brondino 2015
Meiri 2009	Roux 2015
Parellada 2017	De Crescenzo 2020, Fraguas 2019
Voigt 2014	Agostoni 2017, Brondino 2015, Cheng 2017, De Crescenzo 2020, Fraguas 2019, Gogou 2017, Horvath 2017, Li 2017a, Monteiro 2020, Sathe 2017, Williamson 2017
**Vitamin D**	
Azzam 2015	Mazahery 2016, Gillberg 2017, Focker 2017
Feng 2016	Mazahery 2016, Focker 2017
Kerley 2017	Li 2017b
Saad 2016 (retracted)	Gillberg 2017, Focker 2017, Li 2017a, Li 2017b, Mazahery 2016
Ucuz 2015	Gillberg 2017, Focker 2017, Mazahery 2016
**Vitamine B6 (+ Mg)**	
Barthelemy 1980	Kleijnen 1991
Findling 1997	Brondino 2015, Fraguas 2019, Gogou 2017, Li 2017b, Nye 2002
Jonas 1984	Kleijnen 1991
Lelord 1981	Li 2017b
Tolbert 1993	Brondino 2015, Gogou 2017, Li 2017b, Nye 2002
Rimland 1978	Fraguas 2019, Kleijnen 1991
Martineau 1985	Gogou 2017, Kleijnen 1991, Li 2017b
**Folic acid**	
Frye 2016	Fraguas 2019, Li 2017a, Li 2017b
**Folic acid + B12**	
Frye 2013	Brondino 2015
**L-carnitine**	
Geier 2011	Malaguarnera 2019, Sathe 2017, Williamson 2017
Fahmy 2013	Malaguarnera 2019, Sathe 2017, Williamson 2017
Goin-Kochel 2019	Malaguarnera 2019
**Vitamin B12**	
Bertoglio 2010	Brondino 2015, Fraguas 2019, Gogou 2017, Li 2017a, Sathe 2017, Williamson 2017
Hendren 2016	Li 2017a, Li 2017b, Monteiro 2020, Sathe 2017, Williamson 2017
**Vitamin A**	
Guo 2018	Monteiro 2020
**Amino acids**	
Wink 2016	Gogou 2017
Minshavi 2016	Gogou 2017
Nikoo 2015	Gogou 2017
Ghanizadeh 2013	Gogou 2017
Hardan 2012	Gogou 2017
Posey 2004	Gogou 2017
Kern 2001	Gogou 2017
**Vitamin C**	
Dolske 1993	Brondino 2015, Fraguas 2019, Gogou 2017
**Inositol**	
Levine 1997	Gogou 2017

**Multivitamins**	
Adams 2011	Brondino 2015, Fraguas 2019
Adams 2004	Fraguas 2019, Monteiro 2020, Murza 2010
**Flavanoids**	
Taliou 2013	Brondino 2015
**L-carnosine**	
Chez 2002	Brondino 2015

References

1. Agostoni, C., et al., *The Role of Omega-3 Fatty Acids in Developmental Psychopathology: A Systematic Review on Early Psychosis, Autism, and ADHD.* International journal of molecular sciences, 2017. **18**(12).
2. Bent, S., K. Bertoglio, and R.L. Hendren, *Omega-3 fatty acids for autistic spectrum disorder: a systematic review.* Journal of autism and developmental disorders, 2009. **39**(8): p. 1145-1154.
3. Bostock, E.C.S., K.C. Kirkby, and B.V.M. Taylor, *The current status of the ketogenic diet in psychiatry.* Frontiers in Psychiatry, 2017. **8**.
4. Brondino, N., et al., *Complementary and Alternative Therapies for Autism Spectrum Disorder.* Evidence-Based Complementary and Alternative Medicine, 2015.
5. Buie, T., *The relationship of autism and gluten.* Clinical therapeutics, 2013. **35**(5): p. 578-583.
6. Castro, K., et al., *Effect of a ketogenic diet on autism spectrum disorder: A systematic review.* Research in Autism Spectrum Disorders, 2015. **20**: p. 31-38.
7. Cheng, Y.S., et al., *Supplementation of omega 3 fatty acids may improve hyperactivity, lethargy, and stereotypy in children with autism spectrum disorders: a meta-analysis of randomized controlled trials.* Neuropsychiatric disease and treatment, 2017. **13**: p. 2531-2543.
8. Christison, G.W. and K. Ivany, *Elimination diets in autism spectrum disorders: any wheat amidst the chaff?* Journal of developmental and behavioral pediatrics, 2006. **27**(2 Suppl): p. S162-171.
9. De Crescenzo, F., et al., *Impact of polyunsaturated fatty acids on patient-important outcomes in children and adolescents with autism spectrum disorder: a systematic review.* Health and Quality of Life Outcomes, 2020. **18**(1): p. 12.
10. Focker, M., et al., *Vitamin D and mental health in children and adolescents.* European child & adolescent psychiatry, 2017. **26**(9): p. 1043-1066.
11. Fraguas, D., et al., *Dietary Interventions for Autism Spectrum Disorder: A Meta-analysis.* Pediatrics, 2019. **144**(5): p. 1-14.
12. Gillberg, C., et al., *The role of cholesterol metabolism and various steroid abnormalities in autism spectrum disorders: A hypothesis paper.* Autism Research, 2017. **10**(6): p. 1022-1044.
13. Gogou, M. and G. Kolios, *The effect of dietary supplements on clinical aspects of autism spectrum disorder: A systematic review of the literature.* Brain & development, 2017. **39**(8): p. 656-664.
14. Gogou, M. and G. Kolios, *Are therapeutic diets an emerging additional choice in autism spectrum disorder management?* World journal of pediatrics, 2018. **14**(3): p. 215-223.
15. Horvath, A., J. Lukasik, and H. Szajewska, *Omega-3 Fatty Acid Supplementation Does Not Affect Autism Spectrum Disorder in Children: A Systematic Review and Meta-Analysis.* The Journal of nutrition, 2017. **147**(3): p. 367-376.
16. Hurwitz, S., *The Gluten-Free, Casein-Free Diet and Autism Limited Return on Family Investment.* Journal of Early Intervention, 2013. **35**(1): p. 3-19.
17. James, S., P. Montgomery, and K. Williams, *Omega-3 fatty acids supplementation for autism spectrum disorders (ASD).* The Cochrane database of systematic reviews, 2011(11): p. Cd007992.

18. Kleijnen, J. and P. Knipschild, *Niacin and vitamin B6 in mental functioning: a review of controlled trials in humans.* Biological psychiatry, 1991. **29**(9): p. 931-941.
19. Kraeuter, A.K., R. Phillips, and Z. Sarnyai, *Ketogenic therapy in neurodegenerative and psychiatric disorders: From mice to men.* Progress in Neuro-Psychopharmacology and Biological Psychiatry, 2020. **101 (no pagination)**(109913).
20. Li, Y.J., et al., *Dietary Supplement for Core Symptoms of Autism Spectrum Disorder: Where Are We Now and Where Should We Go?* Frontiers in Psychiatry, 2017. **8**: p. 155.
21. Li, Y.J., Y.M. Li, and D.X. Xiang, *Supplement intervention associated with nutritional deficiencies in autism spectrum disorders: a systematic review.* European journal of Nutrition, 2017.
22. Malaguarnera, M. and O. Cauli, *Effects of L-Carnitine in Patients with Autism Spectrum Disorders: Review of Clinical Studies.* Molecules, 2019. **24**(23): p. 10.
23. Mari-Bauset, S., et al., *Evidence of the Gluten-Free and Casein-Free Diet in Autism Spectrum Disorders: A Systematic Review.* Journal of Child Neurology, 2014. **29**(12): p. 1718-1727.
24. Mazahery, H., et al., *Vitamin D and Autism Spectrum Disorder: A Literature Review.* Nutrients, 2016. **8**(4): p. 236.
25. Millward, C., et al., *Gluten- and casein-free diets for autistic spectrum disorder.* The Cochrane Database of Systematic Reviews, 2004(2): p. Cd003498.
26. Millward, C., et al., *Gluten- and casein-free diets for autistic spectrum disorder.* The Cochrane Database of Systematic Reviews, 2008(2).
27. Monteiro, M.A., et al., *Autism Spectrum Disorder: A Systematic Review About Nutritional Interventions.* Revista paulista de pediatria, 2020. **38**: p. e2018262.
28. Mulloy, A., et al., *Gluten-free and casein-free diets in the treatment of autism spectrum disorders: A systematic review.* Research in Autism Spectrum Disorders, 2010. **4**(3): p. 328-339.
29. Mulloy, A., et al., *Addendum to "gluten-free and casein-free diets in treatment of autism spectrum disorders: A systematic review".* Research in Autism Spectrum Disorders, 2011. **5**(1): p. 86-88.
30. Murza, K.A., et al., *Vitamin B-6-magnesium treatment for autism: the current status of the research.* Magnesium Research, 2010. **23**(2): p. 115-117.
31. Nye, C. and A. Brice, *Combined vitamin B6-magnesium treatment in autism spectrum disorder.* The Cochrane database of systematic reviews, 2002(4): p. Cd003497.
32. Piwowarczyk, A., et al., *Gluten- and casein-free diet and autism spectrum disorders in children: a systematic review.* European Journal of Nutrition, 2018. **57**(2): p. 433-440.
33. Roux, C.l., *Use of omega-3 for improving behavioural outcomes in autism spectrum disorder in children: A review of the literature.* Australian Journal of Herbal Medicine, 2015. **27**(3): p. 105-110.
34. Sathe, N., et al., *Nutritional and Dietary Interventions for Autism Spectrum Disorder: A Systematic Review.* Pediatrics, 2017. **139**(6).
35. Williamson, E., et al., *AHRQ Comparative Effectiveness Reviews.* Medical Therapies for Children With Autism Spectrum Disorder-An Update, 2017.
