# Supplementary material for: A Scoping Literature Review of the Relation between Nutrition and ASD Symptoms in Children
Source: Nutrients. 2022 Mar 26;14(7):1389. doi: 10.3390/nu14071389 (PMC9003544; doi:10.3390/nu14071389)
Supplement: Supplementary file 1 [file nutrients-14-01389-s001.zip › File S3_Tests_used_per_study.pdf]

Supplementary File S3: Diagnosis and effect of treatment questionnaires used.

Table S1. Diagnosis and effect of treatment questionnaires used in studies included in meta-analysis and systematic reviews.

**GFCF diet**	**Diagnosis tool**	**Diagnosis by whom**	**Effect treatment questionnaire/tool**	**By whom**
Cade 2000			Self-constructed questionnaire	Physician and teacher
Elder 2006	DSM-IV and ADI-R	NR (DSM-IV), researcher (ADI-R)	CARS<br>ADI-R<br>ECOS<br>Direct observation	CARS: evaluation team<br>ADI-R: caregivers<br>ECOS:?<br>Direct observation: trained coder
Ghalichi 2016	ADI-R	Psychologist	GARS	GARS: psychiatrist
Goodwin 1971	NR	At Albany Medical Center or State Hospital at Marc	Behaviour evaluation	Interviewer
Hyman 2016	DSM-IV, ADI-R and ADOS	A large, tertiary care developmental and behavioral pediatrics Clinic (DSM-IV), researchers (ADI-R and ADOS)	Ritvo-Freeman Real Life Rating Scales, Conners Abbreviated Rating Scale	Ritvo-Freeman: observer<br>Connor: Parents, instructor and research assistant.
Johnson 2011	DSM-IV, ADOS and observation	Two clinicians	Mullen Scales<br>CBCL<br>Direct Behaviour Observation Measure during ADOS	Mullen: ?<br>CBCL: ?<br>Direct observation: ?
Knivsberg 2002	NR	NR	DIPAB	DIPAB: ?
Knivsberg 2003	NR	By professional in the field of child psychiatry/neurology	DIPAB	DIPAB: parent interviewed by researcher.
Knivsberg 1990	Full text not available to authors	Full text not available to authors	C-Raven<br>Tajford Observation Scheme<br>DIPAB	Full text not available to authors
Knivsberg 1995 (same study as Knivsberg 1990)	DSM-III	NR	Tajford Observation Scheme<br>DIPAB	DIPAB: interview with parents<br>Tajford: teacher

Lucardelli 1995	DSM-III	NR	BSE	BSE: ?
Navarro 2015	DSM-IV, ADI-R, ADOS, clinical interview, observation and record review	psychologist	ABC<br>CPRS-R<br>CBCL	ABC: parents<br>CPRS-R: parents<br>CBCL: parents
Patel 2007	NR	Institutions, neurologist or developmental physicians	assessments of motor, behavioural, and educational capabilities<br>Subjective changes in symptoms of autism and ADHD	Parents, teachers and physician<br>Parents and children
Pedersen 2014 (same study as Whiteley 2010)	ICD-10	At the Center for Autisme or other child psychiatric clinics	ADOS<br>VABS<br>GARS<br>ADHD-RS	ADOS: ?<br>VABS: parents<br>GARS: ?<br>ADHD-RS: parents
Pusponegoro 2015	DSM-IV	principal investigator and/or two child psychologists	PDBBI	PDBBI: parents guided by psychologist.
Reichelt 1990	Full text not available to authors	Full text not available to authors	Behaviour questionnaire	Full text not available to authors
Seung 2007<br>Same study as Elder 2006	DSM-IV, ADI-R	NR (DSM-IV), researcher (ADI-R)	X	X
Whiteley 1999	DSM-IV and/or ICD-10	clinicians	BSE<br>PASS	BSE: parent and teacher<br>PASS: parents
Whiteley 2010 (same study as Pedersen 2014)	ICD-10	At the Center for Autisme or other child psychiatric clinics	ADOS<br>VABS<br>GARS<br>ADHD-RS	ADOS: ?<br>VABS: parents<br>GARS: ?<br>ADHD-RS: parents
**GFCF and ketogenic diet**				
El-Rashidy 2017	DSM-5	senior psychologists	CARS<br>ATEC	CARS: parents via interview<br>ATEC:?
**Ketogenic diet**				
Evangeliou 2003	CARS	Psychiatrist	CARS	CARS: psychiatrist
Lee 2018	NR	Pediatric neurologist	ADOS	ADOS: examiner

			CARS	CARS: clinician
Spilioti 2013	DSM-IV	NR	detailed clinical and psychiatric examination	?
**Chanyi diet**				
Chan 2012	DSM-IV and ADI-R	Clinical psychologist	Neuropsychological assessments<br>ATEC<br>Repetitive/disinhibitory behaviours questionnaire	Neuropsychological: test<br>ATEC: parents<br>Parents
**Camel milk**				
Al-Ayadhi 2013	DSM-IV	NR	CARS<br>Wing Subgroups Questionnaire	CARS: ?<br>WSQ: parents
Bashir 2014	DSM-IV	NR	ADOS<br>CARS	ADOS:?<br>CARS: psychiatrist
**Omega-3 fatty acid**				
Amminger 2007	DSM-IV, ADI-R, ADOS	NR	ABC	ABC: two clinicians
Bell 2004	NR	NR	Unstructured parent report	Parent
Bent 2011	ADOS, SCQ, DSM-IV	Clinician	ABC<br>SRS<br>BASC<br>CGI-I	ABC: parent<br>SRS: parent<br>BASC: parents<br>CGI-I: clinician
Bent 2014	ASD diagnosis reported by parents and SCQ	NR	ABC<br>CGI<br>SRS	ABC: Parent and teacher<br>CGI: Parent<br>SRS: Parent
Johnson 2010	DSM-IV and ADOS	NR	CBCL,<br>Direct Behaviour Observation Measure during ADOS	CBCL:?<br>Behaviour: clinician
Mankad 2015	Confirmed diagnoses of ASD	NR	PDDBI<br>BASC<br>VABS-II,<br>CGI- I.	PDDBI: parents<br>BASC: parents<br>VABS-II: parents?<br>CGI:-I clinician

Mazahery 2019	DSM-5	Developmental paediatrician	ABC	ABC: parent
Meguid 2008	DSM-IV and CARS	NR	CARS	CARS: parents?
Meiri 2009	DSM-IV	Psychiatrist	CARS<br>CGI<br>CPRS<br>ATEC	CARS: clinician<br>CGI: clinician<br>CPRS: clinician<br>ATEC: clinician
Parellada 2017	DSM-IV and ADOS.	Child psychiatrist	SRS<br>CGI-S	SRS: parent<br>CGI-S: clinician?
Voigt 2014	DSMI-IV and CARS	Paediatrician	CGI-I<br>CDI<br>ABC<br>BASC	CGI-I: parent and investigator<br>ABC: parent<br>CDI: parents<br>BASC: parents and teachers
**Vitamin D**				
Azzam 2015	DSM-IV	Child psychiatrist	CARS<br>VABS<br>ATEC	CARS: clinician<br>VABS: clinician<br>ATEC: clinician
Feng 2016	DSM-IV and ADOS	Paediatricians	CARS<br>ABC	CARS: rehabilitation doctor<br>ABC: parent interview
Kerley 2017	ADOS or DSM or Diagnostic Instrument for Social and Communication Disorder	Clinician	ABC<br>DDCGAS<br>SRS	ABC: parent<br>DDCGAS: clinician<br>SRS: parent
Saad 2016 (retracted)			X	
Ucuz 2015	DSM-IV, Denver Developmental Screening test, ADSI	Child and adolescent psychiatrist	ABC<br>CBCL<br>DDST<br>ADSI	ABC: family<br>CBCL: family<br>DDST: psychiatrist<br>ADSI: psychiatrist
**Vitamine B6 (+ Mg)**				

Barthelemy 1980	Original text in French	Original text in French	X	Original text in French
Findling 1997	DSM-III	child and adolescent psychiatrist and child neurologist	CARS<br>CGI<br>Children's Psychiatric Rating Scale<br>NIMH Global Obsessive Compulsive Scale<br>CPRS<br>CTRS	CARS: clinician?<br>CGI clinician?<br>Children's Psychiatric Rating Scale: clinician?<br>NIMH Global Obsessive Compulsive Scale: clinician?<br>CPRS: parents<br>CTRS: teachers
Jonas 1984	Original text in French	Original text in French	Original text in French	Original text in French
Lelord 1981	NR	NR	Bretonneau II Clinical Scale<br>Rimland E2 scale	Clinician?
Tolbert 1993	DSM-III	Two child psychiatrist	Ritvo-Freeman Real Life Rating Scale for Autism	Rater
Rimland 1978	NR	NR	Narrative notes by parents and teachers<br>Individually developed target symptom checklist.	Parents and teachers
Martineau 1985	DSM-III	NR	BSE	Two independent raters
**Folic acid**				
Frye 2016	gold-standard diagnostic instrument such as the ADOS and/or ADI-R and/or the state of Arkansas diagnostic standard, defined as	Diverse, principal investigator (ADI-R)	OACIS<br>VABS<br>ABC<br>SRS<br>BASC<br>AIM<br>ASQ	OACIS: observer<br>VABS: interview with caretaker<br>ABC: parents and teachers<br>SRS: parents and teachers<br>BASC: parents and teachers<br>AIM: parents<br>ASQ: parents

	agreement of a physician, psychologist and speech therapist; and/or (iii) DSM diagnosis by a physician along with standardized validated questionnaires and ADI-R			
**Folic acid + B12**				
Frye 2013	DSM-IV	NR	VABS	VABS: parent interview
**L-carnitine**				
Geier 2011	NR	NR	CARS<br>ATEC<br>CGI	CARS: investigator<br>ATEC: parents<br>CGI: investigator
Fahmy 2013	NR	NR	CARS	CARS: investigator
Goin-Kochel 2019	ADOS and ADI-R	Clinician	SCQ<br>PDDBI<br>AIM<br>ABC<br>CGI	SCQ: parents<br>PDDBI: parents<br>AIM: parents<br>ABC: parents<br>CGI: clinician
**Vitamin B12**				
Bertoglio 2010	DSM-IV, ADOS and ADI-R	Psychologist	PIA-CV<br>CGI-I<br>CARS<br>ABC<br>CBCL	Clinical assessments were administered by a licensed psychologist or an experienced clinician trained to reliability
Hendren 2016	ADI-R, ADOS, clinical review	child and adolescent psychiatrist	CGI-I<br>ABC<br>SRS	CGI-I: clinician observer<br>ABC: parent/caregiver<br>SRS: parent/caregiver
**Vitamin A**				

Guo 2018	DSM-5	Developmental paediatricians and psychologists	ABC<br>CARS<br>GDS	ABC:?<br>CARS:?<br>GDS: ?
**Amino acids**				
Wink 2016	DSM-IV, ADI-R	Study physician	CGI-I<br>CGI-S<br>SRS<br>ABC<br>VABS	CGI-I: study physician<br>CGI-S: parents?<br>SRS: caregiver<br>ABC: parent<br>VABS: caregiver interview
Minshavi 2016	ADOS, ADI-R, DSM-IV	NR	SRS<br>VABS<br>ABC<br>CGI-I<br>TSSA	SRS: teacher<br>VABS: interview with parents/caregivers<br>ABC: parent<br>CGI-I: clinician<br>TSSA: parents and participant
Nikoo 2015	DSM-IV, ADI-R, ABC-C	Child psychiatrist	ABC	ABC: rater with input from parents
Ghanizadeh 2013	DSM-IV, ADI-R	Child and adolescent psychiatrist	ABC	ABC: a resident of Psychiatry
Hardan 2012	DSM-IV, ADI-R and/or ADOS and expert clinical evaluation	NR	ABC<br>RBS-R<br>SRS<br>CGI-S<br>CGI-I	ABC:?<br>RBS-R: ?<br>SRS: parents<br>CGI-S: clinician<br>CGI-I: clinician
Posey 2004	DSM-IV and ADI-R	NR	CGI<br>SRS<br>Children's Yale-Brown Obsessive Compulsive Scale<br>ABC	CGI: ?<br>SRS:?<br>Yale-Brown: ?<br>ABC: parents
Kern 2001	DSM-IV	Authors	VABS<br>ABC	VABS: interviewing parents<br>ABC: examiner and parents
**Vitamin C**				
Dolske 1993	DSM-III	Two child psychiatrists	Ritvo-Freeman Real Life Rating Scale	Rater
**Inositol**				

Levine 1997	DSM-III	NR	CARS<br>CGI<br>CTRS<br>CPRS	CARS: ?<br>CGI:?<br>CTRS: teacher<br>CPRS: parent
**Multivitamins**				
Adams 2011	Diagnosis of ASD	psychiatrist or similar professional	PDDBI<br>ATEC<br>SAS<br>PGI	PDDBI: parent<br>ATEC: parent<br>SAS: parent<br>PGI: parent
Adams 2004	Diagnosis of ASD	Psychiatrist or developmental paediatrician	CGI	CGI: parents
**Flavanoids**				
Taliou 2013	DSM-IV and ADOS	At a psychiatric clinic for ASD.	VABS<br>ABC<br>ATEC<br>CGI-I	VABS: parent interview<br>ABC: ?<br>ATEC: ?<br>CGI-I: parents, primary clinician, trainer
**L-carnosine**				
Chez 2002	DSM-IV (not diagnosed in study)	NR	CARS<br>GARS<br>CGI-C	CARS: parents<br>GARS: parents<br>CGI-C: parents

ABC: The Aberrant Behavior Checklist Scale; ABC-C= The Aberrant Behavior Checklist Scale Community; ADI-R: Autism Diagnostic Interview- Revised; ADHD-RS: ADHD rating Scale -IV; ADOS: Autism Diagnostic Observation Scale; ADSI = Ankara Developmental Screening Inventory; AIM: The Autism Impact Measure; ASQ: Autism Spectrum Quotient; ATEC: Autism Treatment Evaluation Checklist; BASC: Behavior Assessment System for Children; BSE: Behavioral Summarized Evaluation; CARS: childhood autism rating scale; CBCL: child behaviour checklist; CDI: Communicative Development Inventory; CGI-(C): clinical global impression scale (change);CGI-I: clinic global impression scale – improvement; CGI-S: clinical global impression severity; CPRS(-R): Conners' Parent Rating Scale (revised); CTRS: Conners' Teacher Rating Scale; DDCGAS: Developmental Disability-Child Global Assessment Scale; DDST: Denver Developmental Screening test; DIPAB: Diagnosis of Psychotic Behavior in Children; ECOS: Ecological Communication Orientation Scale; GARS: Gilliam Autism Rating Scale; GDS: Gesell Developmental Scale; ICD =International Statistical Classification of Diseases and Related Health Problems; NR = not reported, OACIS: Ohio Autism Clinical Impression Scale; PASS: Parental Satisfaction Survey; PDDBI: Pervasive Developmental Disorder Behavior Inventory; PGI: Parental Global Impressions; PIA-CV: Parent Interview for Autism Clinical Version; RBS-R: The Repetitive Behavior Scale-Revised; SAS: Severity of Autism Scale; SCQ: Social Communication Questionnaire; SRS: Social Responsiveness Scale; TSSA: TRIAD Social Skills Assessment, VABS: The Vineland Adaptive Behavior Scales;

Table S2. Diagnosis and effect of treatment questionnaires used in original studies.

GFCF diet	Diagnosis tool	Diagnosis by whom	Effect treatment questionnaire/tool	By whom
González-Domenech 2020	ICD-10	Child and adolescent psychiatrist	ATEC<br>BSE<br>ABC	child and adolescent psychiatrist/psychologist
Piwowarczyk 2020	DSM-5 or ICD-10	Not in study	ADOS<br>SCQ<br>ASRS<br>VABS	ADOS: a trainee<br>SCQ: parents<br>ASRS: parents<br>VABS: parents
González-Domenech 2019	ICD-10	Child and adolescent psychiatrist	ATEC<br>BSE<br>ABC	ATEC: member research team<br>BSE: member research team<br>ABC: member research team
Hafid 2018	diagnosed with ASD	NR	CARS	CARS: attending physician and the psychomotor therapist
Pennesi 2012	clinical diagnosis of an ASD	NR	Self-developed questionnaire with questions regarding ASD behaviors and social behaviors.	Parents
Harris 2012	diagnosed ASD	NR	CARS	CARS: parents
Amin 2011	clinical child psychiatric sheet DSM-V	NR	CARS	CARS: ?
Nazni 2008	Autistic children	NR	Behavior patterns were elicited via interview schedule	Interview with parents
Al-Ayadhi 2015	DSM-IV	NR	CARS<br>SRS<br>ATEC	CARS: psychologist<br>SRS: psychologist<br>ATEC: psychologist
Hannant 2019	DSM-V and ADOS	Clinician (DSM-V), research team (ADOS)	ASRS	ASRS: parents
Geng 2020	NA	NA	CABS	CABS: parents or guardian

			SDQ	SDQ: parent or guardian
**N-3, N-6 or PUFA supplementation**				
Keim 2018	PDDST<br>BITSEA, 1 question to assess joint attention	Parents	PDDST<br>BITSEA<br>1 question to assess joint attention	PDDST: Parents<br>BITSEA: Parents<br>1 question: Parents
Ooi 2015	DSM-IV	Child psychiatrist	SRS<br>CBCL	SRS: parents<br>CBCL: parents
**Vitamins and minerals**				
Mazahery 2019, 2020	DSM-5	Developmental paediatrician	SRS<br>SPM<br>ABC	SRS: caregiver<br>SPM: caregiver<br>ABC: caregiver
Moradi 2020	DSM-5	Psychologist	GARS	GARS: parents, teachers and observations
Ali 2018	NA	NA	Physician diagnosis of ASD (via ADOS or DSM-IV or DSM-5)<br>ITS	Physician diagnosis: physician<br>ITC: parents
Bittker 2018	Reported by parents	Professional	ASD diagnosis	ASD diagnosis: reported by parents
Jia 2018	DSM-V	NR	ABC<br>CARS	ABC: parent interview<br>CARS: neurodevelopmental paediatrician
Liu 2017	DSM-V	Developmental paediatrician	ABC<br>CARS<br>SRS	ABC: parent interview<br>CARS: parent interview<br>SRS: parent interview
Sun 2016	DMV-IV	Specialist clinician	ABC<br>CARS<br>ATEC<br>PEP-3	ABC: administered by trained nurse<br>CARS: administered by trained nurse<br>ATEC: parents<br>PEP-3: direct testing and observing and caregiver report

Gillberg 1986	DSM-III	Child psychiatrist	ABC<br>Other checklists and questionnaires	ABC: interview with parents by clinician<br>Other: interview with parents by clinician
Fattal-Valevski 2009	DMS-III	Child psychiatrist	CARS<br>M-CHAT	CARS: developmental neuro-psychologist<br>M-CHAT: developmental neuro-psychologist
Antonucci 2017	NR	Child neurologist or developmental psychologist and clinician	CGI-I	CGI-I: clinician
Reynolds 2020	DSM-IV and ADOS	NR	ABC<br>SNAP-IV<br>RBS-R	ABC:?<br>SNAP-IV: ?<br>RBS-R:?
Ramaekers 2019	ADI-R, ADOS, CARS and psychological and psychiatric assessment and observations	NR	CARS	CARS:?
Mehrazad-Saber 2018	DSM-5	Clinician	GARS	GARS: ?
Meguid 2019	DSM-IV, ADI-R and CARS	NR	CARS	CARS:?

ABC: The Aberrant Behavior Checklist Scale; ADI-R: Autism Diagnostic Interview- Revised; ADOS: Autism Diagnostic Observation Scale; ASD: autism spectrum disorder, ATEC: Autism Treatment Evaluation Checklist; BITSEA: Brief Infant Toddler Social and Emotional Assessment; BSE: Behavioral Summarized Evaluation; CARS: childhood autism rating scale; CBCL: child behaviour checklist; CGI-I: clinic global impression scale – improvement; DSM: The Diagnostic and Statistical Manual of Mental Disorders; GARS: Gilliam Autism Rating Scale; ICD: International Statistical Classification of Diseases and Related Health Problems; ITS: Infant-Toddler Checklist; M-CHAT: The Modified Checklist for Autism in Toddlers; NA: not appropriate; NR: not reported; PDDST: Pervasive Developmental Disorders Screening Test, PEP-3: Psychoeducational Profile; SNAP-IV: Swanson, Nolan, and Pelham-IV; RBS-R: The Repetitive Behavior Scale-Revised; SPM: Sensory Processing Measure. SRS: Social Responsiveness Scale.
